# Assessing Evolutionary Divergence in Genome‐Wide MADS‐Box Genes and Expression Profiles Between *Toona ciliata* and *Toona sinensis*

**DOI:** 10.1002/ece3.72328

**Published:** 2025-10-13

**Authors:** Xiao‐Han Liu, Yu Xiao, Yan‐Wen Lv, Zi‐Yun Wang, Chao Wu, Hui Xie, Xin‐Sheng Hu

**Affiliations:** ^1^ College of Forestry and Landscape Architecture South China Agricultural University Guangzhou China; ^2^ Guangdong Key Laboratory for Innovative Development and Utilization of Forest Plant Germplasm Guangzhou China

**Keywords:** MADS‐box gene family, plant flowering, purifying selection, *Toona ciliata*, *Toona sinensis*

## Abstract

*Toona ciliata* and *Toona sinensis*, two economically important timber species in China, exhibit interspecific variation in floral traits that are related to mating systems. The MADS‐box genes play a crucial role in floral patterning of angiosperms. Here we identified 97 and 75 MADS‐box genes at the genome scale in *T. ciliata* and *T. sinensis* , respectively, which were classified into Type I and Type II groups. Genes within the same subfamily exhibited high homology and conserved motifs. Promoter analysis revealed cis‐acting elements related to growth, light response, hormone signaling, and stress response. Gene duplication was prevalent, with several genes undergoing multiple duplications. Most MADS‐box genes were under purifying selection between *T. ciliata* and *T. sinensis* , while eight orthologous genes in the AGL32, Mβ, MIKC*, AG/STK, and AP1/FUL subfamilies were under weakly positive selection. Transcriptomic analysis showed high gene expression in SEP, AP1/FUL, AG/STK, PI, and AP3 subfamilies, highlighting their roles in floral development. A significantly negative correlation occurred between the evolutionary rate ($K_{a}/K_{s}$) and gene expression level of MADS‐box genes, suggesting evolutionary constraints on highly expressed genes. TWAS (transcription‐wide association study) indicated that some MADS‐box genes were significantly associated with floral traits in *T. sinensis* . The overall findings provide insights into the roles of MADS‐box gene family in evolving interspecific divergence in floral development between *T. ciliata* and *T. sinensis* .

## Graphical abstract

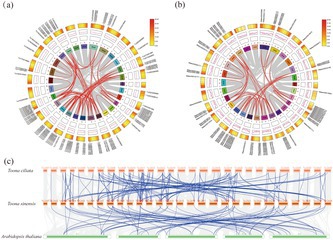

## 1 Introduction

*T. ciliata* and *T. sinensis* are deciduous or semi‐deciduous plants and belong to the *Toona* genus of the Meliaceae family. *T. ciliata* is distributed across tropical and subtropical regions, including India, Malaysia, Indonesia, and southern China. This species is highly valued for its timber properties, such as the bright reddish‐brown heartwood, beautiful grain patterns, and durable, corrosion resistance. As such, it is a preferred material for crafts and furniture and has significant economic value, earning the nickname “Chinese Mahogany” (Cheng and Cui 2010; Edmonds and Staniforth 1998). The species is also used as the medicinal material for hemostasis, antibacteria, and anti‐inflammation (Malairajan et al. 2007; Selvalakshmi et al. 2007). The species is taxonomically divided into five varieties according to leaf and flower characteristics, encompassing *T. ciliata* var. *ciliata*, *T. ciliata* var. *pubescens*, *T. ciliata* var. *yunnanensis*, *T. ciliata* var. *henryi*, and *T. ciliata* var. *sublaxiflora* (Chen et al. 1997), and these varieties are closely related (Lv et al. 2025; Xiao et al. 2023). Owing to overexploitation and low natural regeneration, *T. ciliata* is classified as an endangered species at the second class in China (Fu and Jin 1992; National Forestry and Grassland Administration & Ministry of Agriculture and Rural Affairs 2021). Its genetic conservation has received ever‐increasing concern.

**FIGURE 1:**
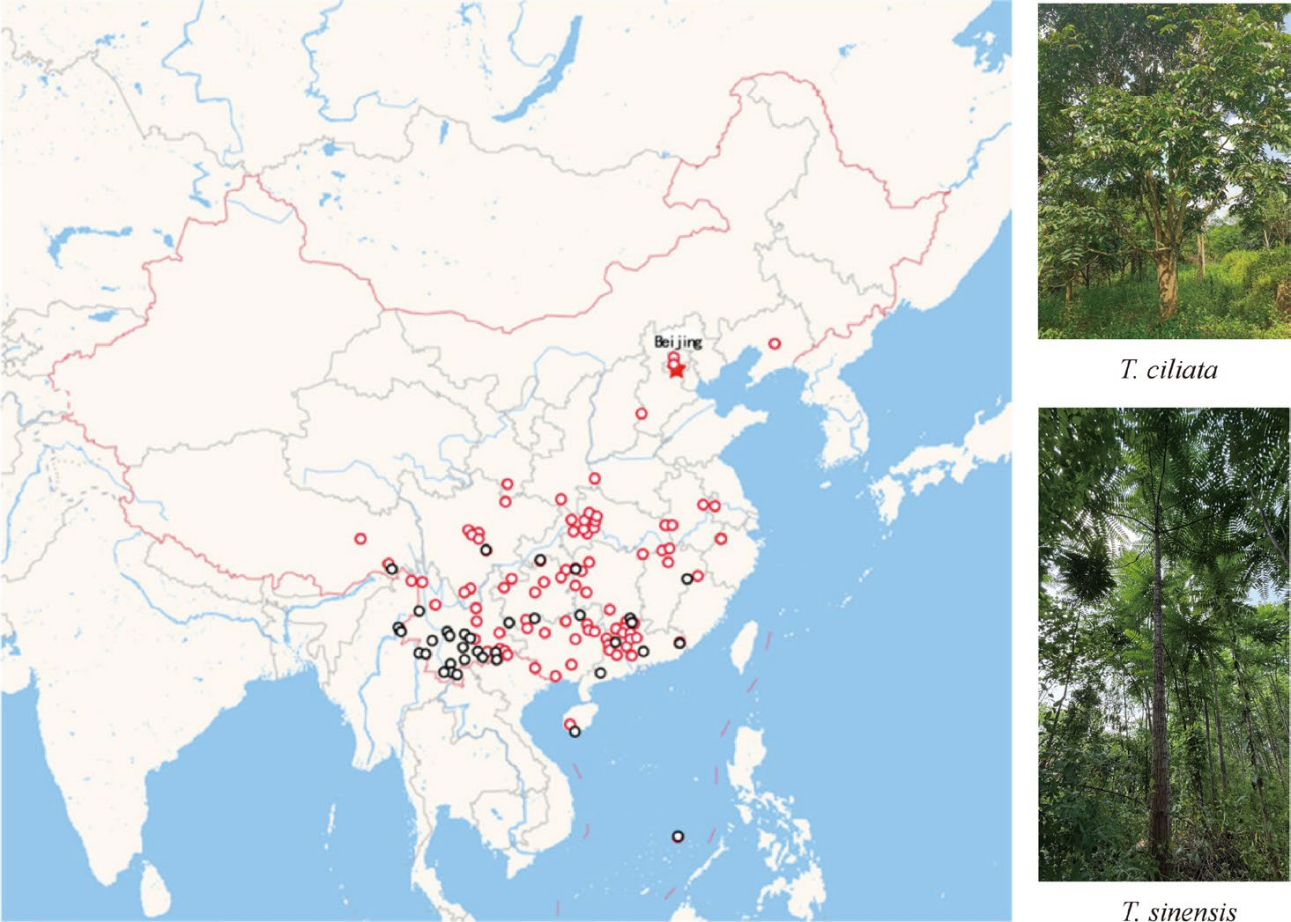
Different ranges in natural distribution between *T. ciliata* (black circles) and *T. sinensis* (red circles) in China. This figure was drawn by synthesizing the distribution map of specimens of the two species from the Chinese Virtual Herbarium website (https://www.cvh.ac.cn/). The distribution of each species can be searched by inputting the species names on the website. Each point in the map represents a record of specimens collected from the corresponding position (Wang, Xiao, He, Li, Lv, et al. 2022).

Unlike *T. ciliata* , *T. sinensis* has been cultivated for more than 2000 years in China. The tree barks are dark brown, and its inflorescences are terminal panicles. Both the young leaves and flowers emit a distinctive fragrance, and the flowering period occurs from June to July. The natural distribution of *T. sinensis* ranges from the southern temperate zone to the northern tropical regions of China (Lu et al. 2001), much wider than that of *T. ciliata* in China (Figure 1). Besides being a source of high‐quality timber, *T. sinensis* is widely used as the material for food sources as well as for traditional Chinese medicine and is a valuable economic species (Hao 2003; Jiao et al. 2019; Liao et al. 2009; Peng et al. 2019). In general, both *T. ciliata* and *T. sinensis* are precious species for timber industrial and medicinal purposes in China (Wang, Xiao, He, Li, Lv, et al. 2022).

One striking feature is that both species initially have perfect flowers in floral development, but as flower development proceeds, some flowers become functionally unisexual, producing different mating systems between them. *T. sinensis* develops a more strictly outcrossing system, whereas *T. ciliata* exhibits a mixed mating system, that is, predominantly outcrossing with selfing and inbreeding (Zhou et al. 2020). There are significant interspecific variations in floral traits (Cheng 2021), and genes associated with interspecific floral differences potentially participate in forming their divergent mating systems (Dart et al. 2012; Goodwillie et al. 2006; Lin and Ritland 1997). The two species were divergent at about 6–25 Mya from common ancestral populations (Wang, Xiao, He, Li, Lv, et al. 2022). The long‐term process of natural selection could evolve their developmental divergence and produce differential expression of genes associated with floral traits. Thus, identifying genes associated with floral traits and assessing their evolutionary mechanisms would provide insights into their roles in forming divergent mating systems.

MADS‐box genes are excellent candidates for studying functional genes associated with floral traits, such as the formation of floral morphology and initialization of flowering time. These genes exhibit similarities in sequence, protein‐coding characteristics, and functional properties (Theißen et al. 1996) and consist of a family of MADS‐box genes. They encode transcription factors that share a common DNA‐binding domain (MADS‐box) and recognize similar target DNA sequences. Formation of this gene family can be traced back to a key gene duplication event, which yielded two major lineages: SRF (serum response factor)‐like (Type I) and MEF2 (myocyte enhancer factor 2)‐like (Type II) (Alvarez‐Buylla et al. 2000). Type I genes encode proteins that possess a core MADS domain (M domain), with a relatively simple structure, and are classified into three subfamilies: Mα, Mβ, and Mγ (Parenicova et al. 2003; Wu et al. 2010). Type II genes, also known as MIKC‐type, encode proteins that, in addition to the MADS conserved domain, contain an intervening domain (I domain), a characteristic keratin‐like domain (K domain), and the least conserved carboxyl‐terminal domain (C domain) (Riechmann and Meyerowitz 1997). The MIKC‐type proteins are further classified into two subfamilies, MIKCC and MIKC*, based on the repetitive exon sequences within the K domain (Chen et al. 2017; Lai et al. 2019), displaying complex structure.

Ample evidence showed the significant effects of the MADS‐box gene family in a wide range of functions in plants (Airoldi and Davies 2012; Smaczniak et al. 2012; Tang et al. 2022; Zhang et al. 2024). For instance, there were 38 Type I genes expressed during female gametophyte and seed development in *Arabidopsis thaliana* (Qiu and Köhler 2022). MIKC* genes (Type II) were expressed during pollen development in both *A. thaliana* and *Oryza sativa* , playing a crucial role in pollen maturation (Liu et al. 2013). MIKC^C^ genes are involved in reproductive processes, such as floral induction and the formation of floral organs. Expression of subfamily genes, such as *SVP*, *FLC*, *SOC1*, *ANR1*, and *AP1*/*FUL*, is influenced by the developmental stages, environmental factors (such as light and temperature), and hormones, which further promote or impede the transition from vegetative to reproductive growth and determine floral organ identity. This regulation enhances plant adaptation to changing environments (Guo et al. 2019; Kennedy and Geuten 2020; Yue et al. 2021; Zhang et al. 2022). Evidence also implies that MADS‐box genes play a conservative and dominant role in both floral development and self‐incompatibility. For instance, *Taraxacum kok‐saghyz* (apomixis) and *T. mongolicum* (self‐incompatibility) exhibited entirely different reproductive modes, where the *MADS‐55* gene in *T. kok‐saghyz* was potentially involved in self‐incompatibility (Chen et al. 2023).

Here we conducted genome‐wide identification of the MADS‐box gene family in *T. ciliata* and *T. sinensis* based on our previous genome sequencing of *T. ciliata* and transcriptomic data of 150 samples of flowers. Since *T. ciliata* is classified into five varieties according to leaf and flower characteristics (Chen et al. 1997), we proceeded to employ two varieties ( *T. ciliata* var. *ciliata* and *T. ciliata* var. *pubescens*) that are closely related (Lv et al. 2025; Xiao et al. 2023) and compared their divergence from *T. sinensis* in expression profiles of the MADS‐box genes. We analyzed phylogenetic relationships among MADS‐box genes, chromosomal mapping of the family members, and the evolutionary processes underlying orthologous MADS‐box genes between species. Besides, TWAS was conducted to identify the MADS‐box genes that were associated with floral traits. By assessing the evolutionary divergence between *T. ciliata* and *T. sinensis* in MADS‐box genes, we elucidated the role of the MADS‐box gene family in maintaining interspecific variation in floral traits and hence in potentially shaping divergent mating systems between *T. ciliata* and *T. sinensis* .

## 2 Results

### 2.1 Identification and Structure of the MADS‐Box Genes

A total of 97 and 75 MADS‐box gene family members were identified from the whole genomes of *T. ciliata* var. *ciliata* and *T. sinensis* , respectively. Analysis of the physicochemical properties of these MADS‐box genes (Table S1) revealed that the coding sequences (CDS) in *T. ciliata* var. *ciliata* ranged from 357 to 1221 bp, while the coding sequences in *T. sinensis* ranged from 255 to 2202 bp. The molecular weights of the corresponding proteins coded by these genes ranged from 13.8 to 45.9 KDa in *T. ciliata* var. *ciliata* and from 9.6 to 83.9 KDa in *T. sinensis* . The isoelectric points (pI) of the proteins ranged from 4.65 to 10.28 for *T. ciliata* var. *ciliata* and from 4.64 to 10.18 for *T. sinensis* . Notably, in *T. ciliata* var. *ciliata*, 25 MADS‐box proteins had the pI below 7, indicating the acidic nature, while 72 proteins had the pI above 7, indicating alkalinity. In *T. sinensis* , 37 MADS‐box proteins exhibited the acidic nature (pI < 7), and 38 proteins were alkaline (pI > 7). Analysis of physicochemical data indicated considerable variations in the properties of MADS‐box gene family members, indicating the complexity and diversity of MADS‐box proteins in both *T. ciliata* var. *ciliata* and *T. sinensis* . Subcellular localization results indicated that these MADS‐box proteins were localized in the nucleus, consistent with their role as transcription factors (Table S1).

**FIGURE 2:**
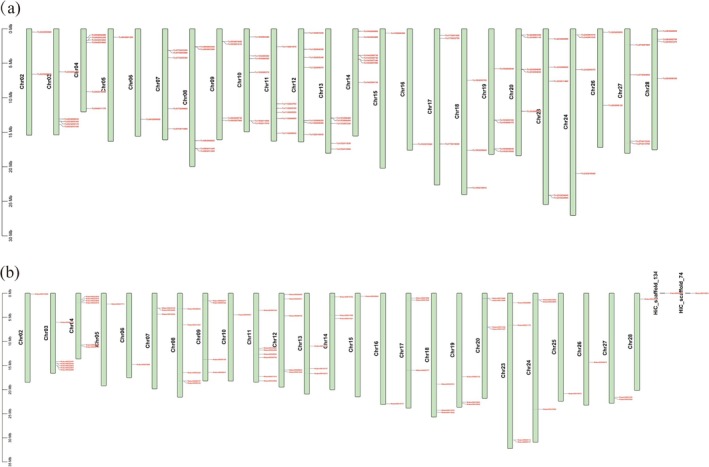
Distribution of MADS‐box gene family members across chromosomes. (a) *T. ciliata* var. *ciliata*; (b) *T. sinensis* . The orange‐red labels indicate the physical positions of MADS‐box genes on each chromosome. Chromosome sizes were scaled down according to their real lengths (Mb).

To investigate the physical distribution of MADS‐box genes on chromosomes, we observed that the MADS‐box genes in *T. ciliata* var. *ciliata* were unevenly distributed across 24 chromosomes, without MADS‐box genes on chromosomes Chr01, Chr21, Chr22, and Chr25 (Figure 2a). Similarly, the MADS‐box genes in *T. sinensis* were unevenly distributed across 25 chromosomes, without MADS‐box genes on chromosomes Chr01, Chr21, and Chr22. Additionally, two MADS‐box genes (Maker00006466 and Maker00010054) from the genome's GFF annotation file were not located on any chromosome (Figure 2b).

### 2.2 Conserved Motifs of MADS‐Box Proteins

**FIGURE 3:**
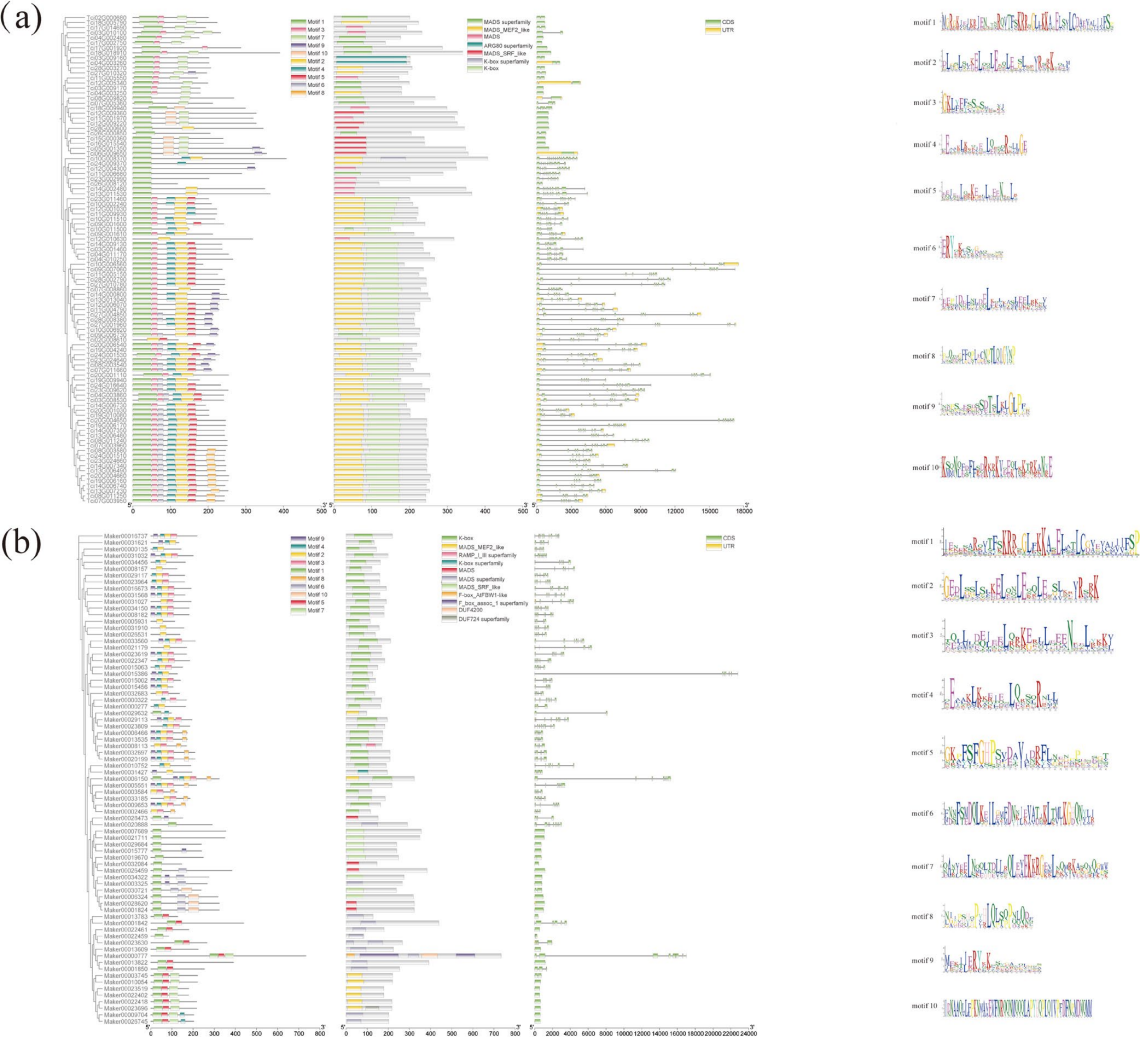
Conserved protein motifs and structure of MADS‐box genes. (a) *T. ciliata* var. *ciliata*; (b) *T. sinensis* . The left panel shows the phylogenetic relationships among proteins encoded by the MADS‐box genes. The middle panels illustrate the distribution of conserved motifs (colored boxes) and exon–intron structure of the corresponding genes. The right panels display sequence logos of the conserved motifs generated by MEME.

To characterize the structure of the MADS‐box gene family members in *T. ciliata* var. *ciliata* and *T. sinensis* , we predicted the conserved motifs within their amino acid sequences. The results showed that the MADS‐box genes in both species encoded proteins containing either the MADS domain or the K‐box domain, with 10 conserved motifs identified and labeled by Motif 1 to Motif 10 (Figure 3). In *T. ciliata* var. *ciliata*, the 97 MADS‐box genes contained 1 to 7 motifs and were distributed diversely, with Motif 1 corresponding to the typical MADS‐box domain and Motif 2 corresponding to the K‐box domain (Figure 3a). In *T. sinensis* , the 75 MADS‐box genes contained 1 to 6 motifs, with a similar diverse distribution; Motif 1 also represented the typical MADS‐box domain, while Motif 4 represented the K‐box domain (Figure 3b). Most genes in both species contained the typical MADS‐box domain, indicating its common role in the gene family, while the distribution of other motifs reflected structural similarities within subfamilies.

To gain a deeper understanding of the structural diversity, similarity, and evolutionary relationships of MADS‐box genes, we further analyzed the arrangement of introns and exons, along with the conserved motifs. The results (Figure 3) indicated that, in *T. ciliata* var. *ciliata*, 15 out of the 97 MADS‐box genes (15.96%) lacked introns, and most genes contained 5 to 8 introns. In *T. sinensis* , 22 out of the 75 MADS‐box genes (29.33%) lacked introns, and most MADS‐box genes contained 4 to 7 introns. A comparison analysis revealed different numbers of introns between the two species, with Type I genes having simpler structures and fewer introns, and Type II genes having more complex structures and more introns.

### 2.3 Cis‐Acting Elements in Promoters of the MADS‐Box Genes

**FIGURE 4:**
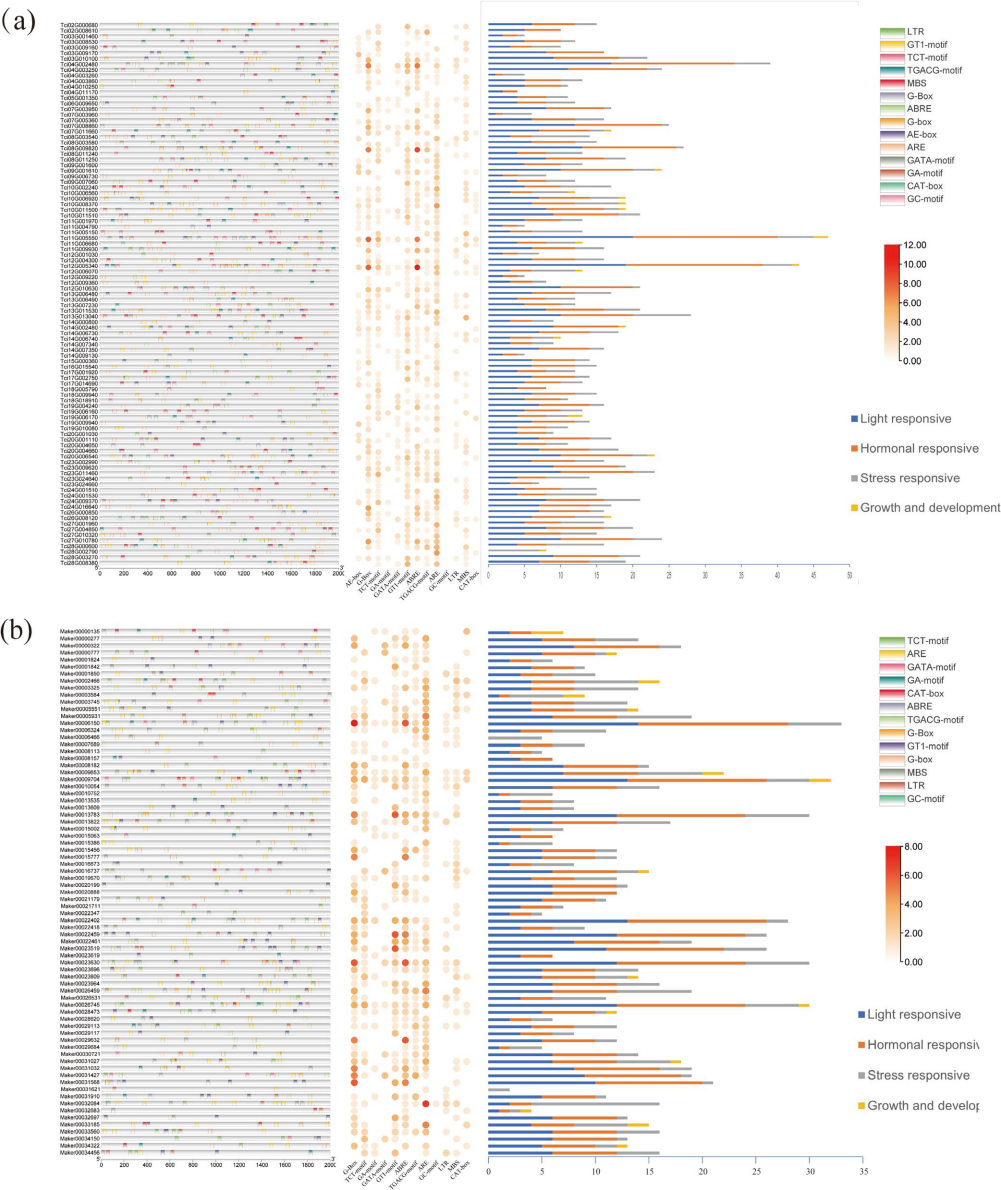
Classification and functions of cis‐acting elements in MADS‐box gene promoters: (a) *T. ciliata* var. *ciliata*; (b) *T. sinensis*. The figure consists of, from left to right, the distribution map of promoter elements, the bubble plot of promoter elements, and the stacked bar chart of different functions.

The distribution of cis‐acting elements in gene promoters is related to potential regulatory mechanisms and functional roles of the genes. Figure 4 shows the cis‐acting elements in promoters of MADS‐box genes in *T. ciliata* var. *ciliata* and *T. sinensis* . The MADS‐box gene promoters in both species contained various elements, including light‐responsive, plant growth, stress‐responsive, and hormone response elements. Among these, the light‐responsive elements were particularly abundant, indicating that light could play a significant role in regulating the expression of MADS‐box genes in *T. ciliata* var. *ciliata* and *T. sinensis* . Additionally, a considerable number of hormone response elements were identified in the promoters of MADS‐box genes in both species. Given the critical roles of plant hormones in flower initiation and organ development, MADS‐box genes in both species could play important roles in flower development and organ formation.

### 2.4 Protein–Protein Interaction Networks of the MADS‐Box Genes

**FIGURE 5:**
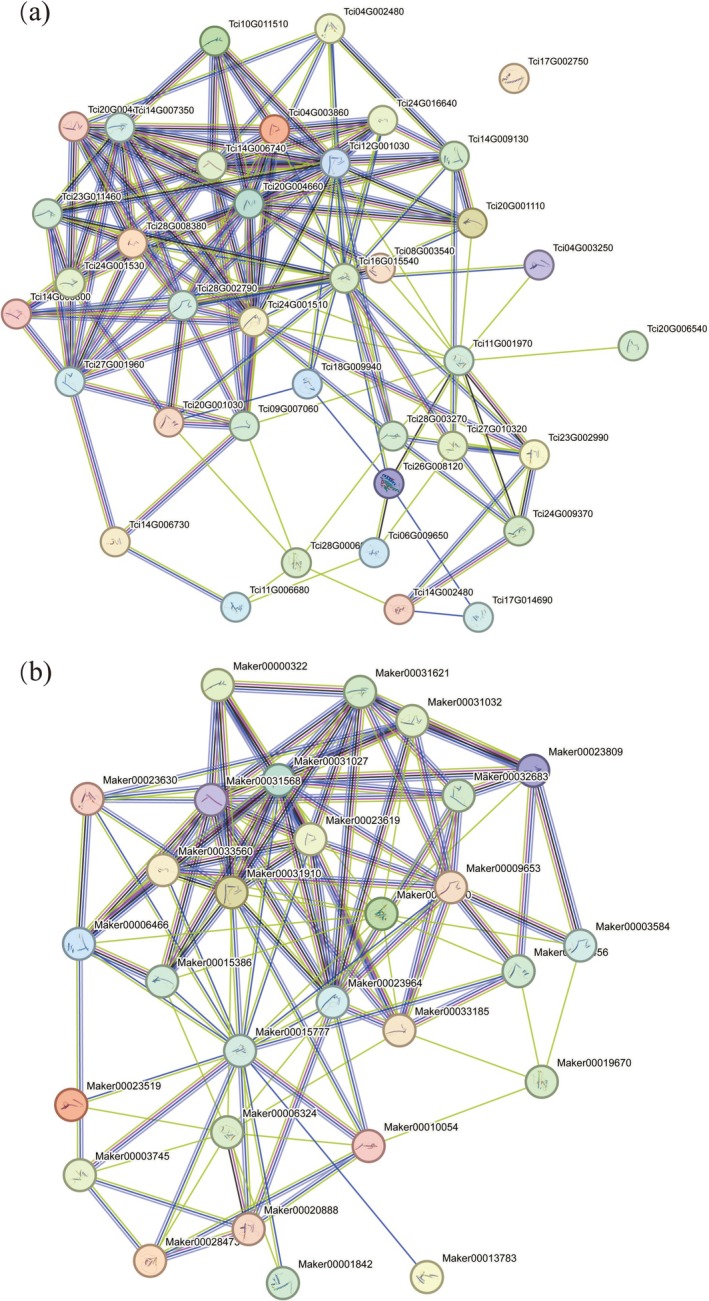
Networks for protein–protein interactions (PPIs) among proteins encoded by MADS‐box genes: (a) *T. ciliata* var. *ciliata*; (b) *T. sinensis* . PPIs were predicted using the STRING database with *A. thaliana* as the reference.

To explore the functional relationships of MADS‐box genes in *T. ciliata* var. *ciliata* and *T. sinensis* , we predicted the protein–protein interaction (PPI) networks using the STRING database, with *A. thaliana* proteins as the reference. The results showed that, in *T. ciliata* var. *ciliata*, all MADS‐box proteins except for Tci17G002750 (a member of the Mα subfamily) exhibited different degrees of interactions with other proteins. Notably, the Tci20G004660 protein from the SEP subfamily was positioned at the center of the network, showing strong interactions with the other gene family members (Figure 5a). In *T. sinensis* , MADS‐box proteins also displayed different levels of interactions, where Maker00031568 and Maker00031027 from the SEP subfamily, Maker00023619 from the AG subfamily, and Maker00031910 from the AP3 subfamily at the network's core demonstrated close interactions with the other gene family members (Figure 5b).

### 2.5 Evolution Mechanisms of the MADS‐Box Genes

**FIGURE 6:**
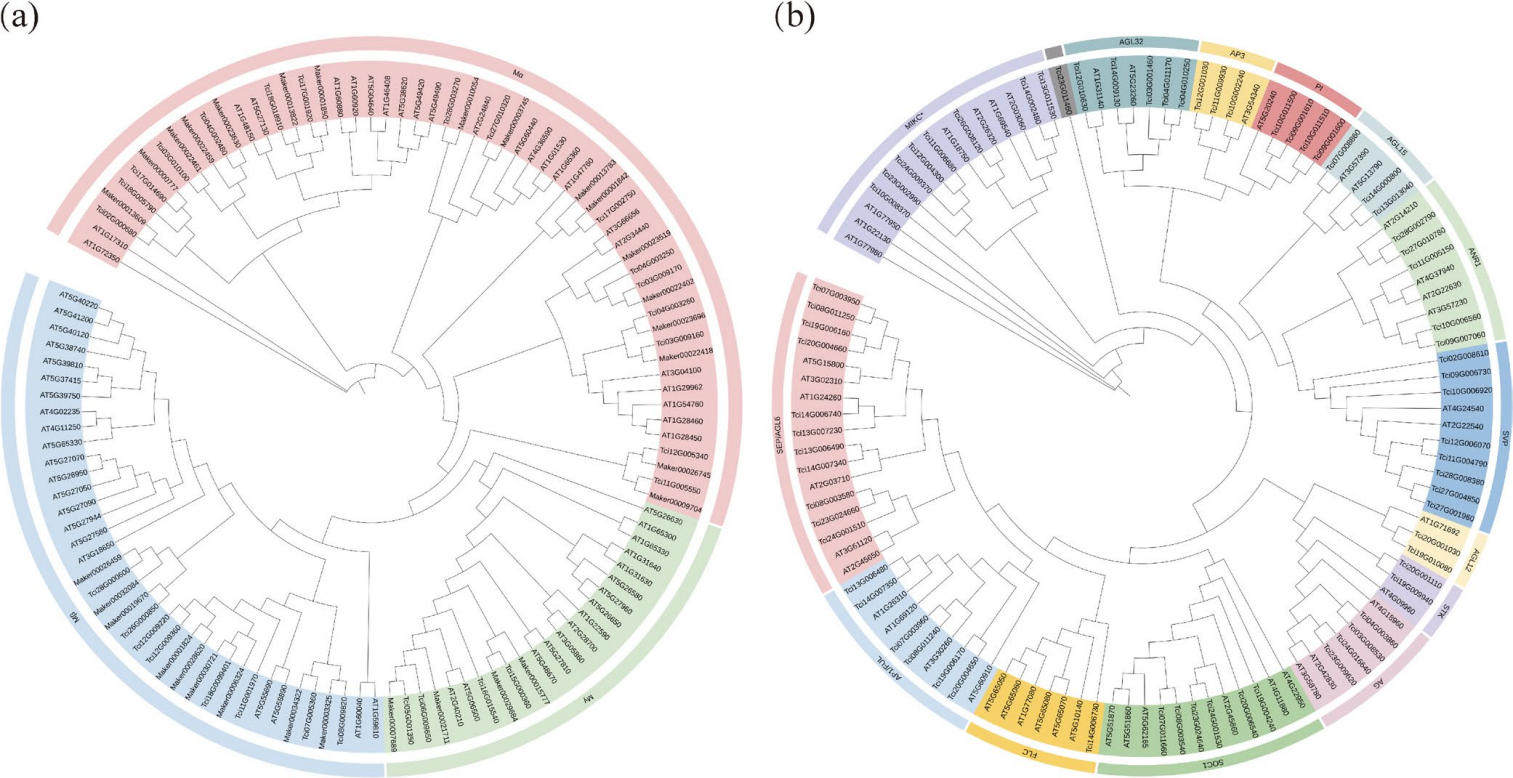
Phylogenetic tree of MADS‐box proteins among *T. ciliata* var. *ciliata*, *T. sinensis* , and *A. thaliana* . (a) Type I MADS‐box proteins; (b) Type II MADS‐box proteins. Subfamilies are indicated in different colors based on their classifications.

Multiple sequence alignments were performed for 97 MADS‐box family members in *T. ciliata* var. *ciliata* and 75 MADS‐box family members in *T. sinensis*. The relevant proteins from *A. thaliana* as the outgroup were employed to construct a phylogenetic tree using IQ‐TREE2. The phylogeny of the MADS‐box family members was separately constructed with Type I and Type II groups. Type I was further divided into three subfamilies: Mα, Mβ, and Mγ (Figure 6a); while Type II was divided into two subgroups, MIKC* and MIKC^C^. The MIKC^C^ group was further subdivided into subfamilies including SEP/AGL6, AP1/FUL, SOC1, FLC, AG/STK, AGL12, ANR1, SVP, AGL15, AP3, PI, and AGL32 (Figure 6b). The MADS‐box family members in *T. ciliata* var. *ciliata* were distributed across all major clades, although the Tci23G011460 gene was not assigned to a specific category. The MADS‐box family members in *T. sinensis* were found in all subfamilies except for FLC, indicating the absence of FLC that was likely related to the unique biological characteristics or environmental adaptation of this species.

**FIGURE 7:**
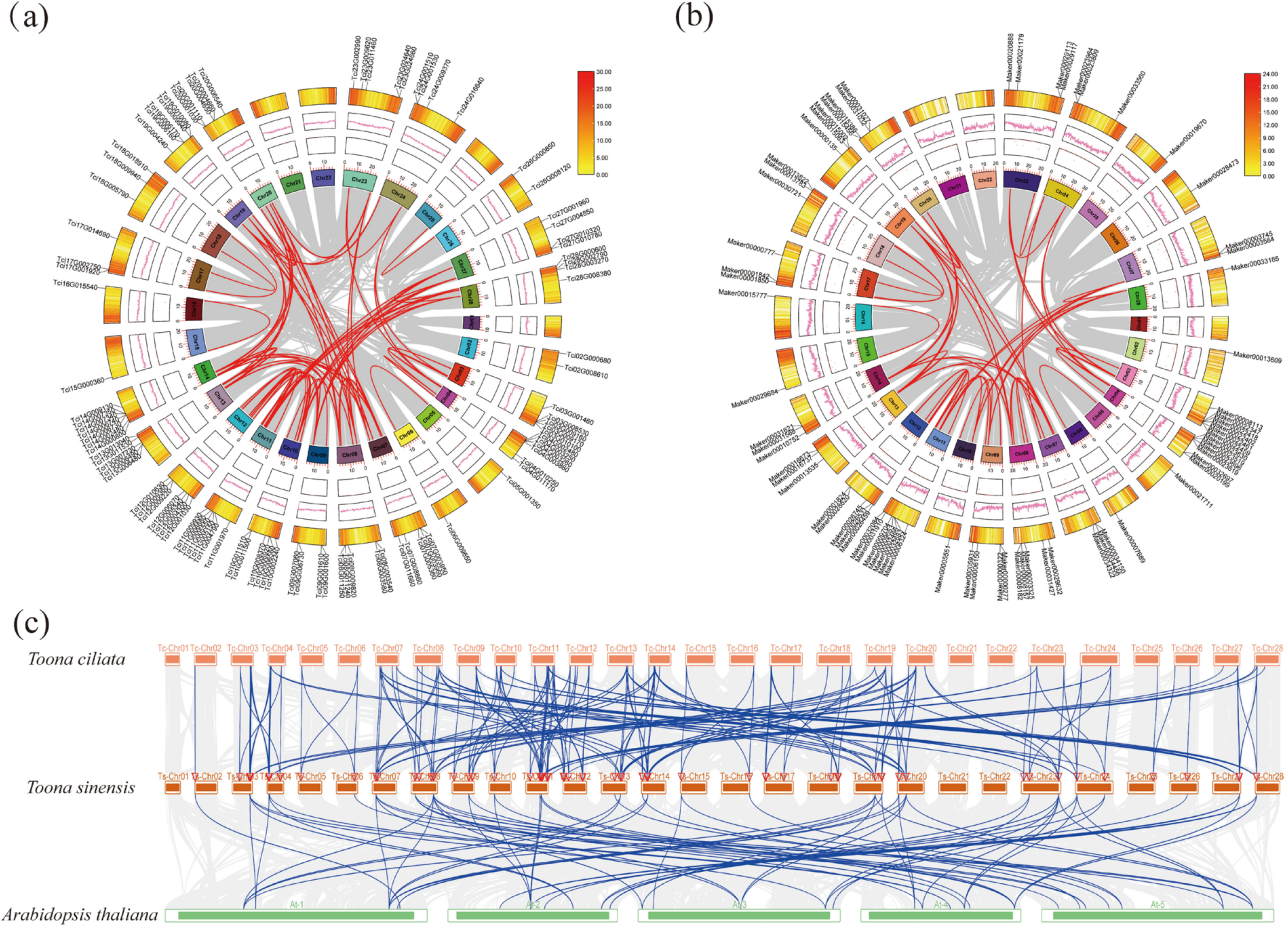
Collinearity analysis of MADS‐box genes in *T. ciliata* var. *ciliata*, *T. sinensis* , and *A. thaliana* . (a) Intragenomic collinearity and duplication events of MADS‐box genes in *T. ciliata* var. *ciliata* (red lines for collinearity between genes); (b) Intragenomic collinearity and duplication events of MADS‐box genes in *T. sinensis* (red lines for collinearity between genes); (c) Interspecific collinearity of MADS‐box genes among *T. ciliata* var. *ciliata*, *T. sinensis* , and *A. thaliana* (blue lines for collinearity between orthologous genes).

Intra‐and interspecific collinearity analyses were conducted to investigate gene duplication events with the MADS‐box genes in *T. ciliata* var. *ciliata*, *T. sinensis* , and *A. thaliana* . The intraspecific collinearity analysis revealed 100 and 63 homologous gene pairs in *T. ciliata* var. *ciliata* (Figure 7a) and *T. sinensis* (Figure 7b), respectively. The MADS‐box gene family in both species underwent different degrees of conserved evolution and gene duplication events. The interspecific synteny analysis revealed 213 homologous gene pairs between *T. ciliata* var. *ciliata* and *T. sinensis* , and 72 homologous gene pairs between *T. sinensis* and *A. thaliana* (Figure 7c). The MADS‐box gene family showed numerous collinearity blocks, indicating multiple duplication events between species. These results indicate that the MADS‐box gene family exhibited high intraspecific conservation, although it underwent frequent interspecific duplication events in the genomes of *T. ciliata* var. *ciliata* and *T. sinensis* .

**FIGURE 8:**
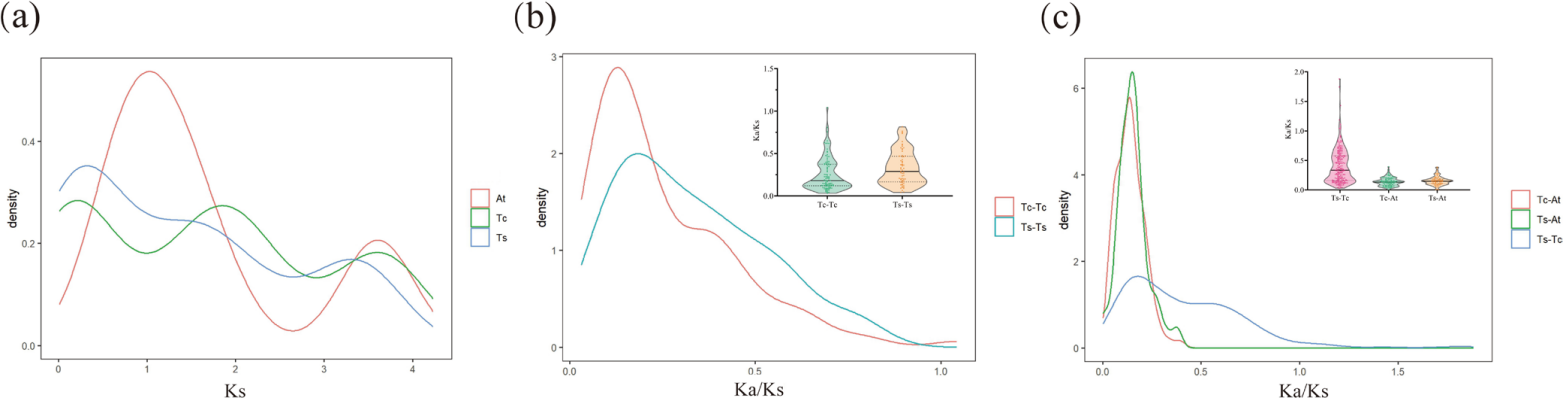
Distribution of synonymous ($K_{s}$) and nonsynonymous ($K_{a}$) substitution rates and their ratio ($K_{a}/K_{s}$) of the MADS‐box genes. (a) $K_{s}$ for the paralogous genes within *T. ciliata* var. *ciliata*, *T. sinensis* and *A. thaliana* ; (b) $K_{a}/K_{s}$ between paralogous members in *T. ciliata* var. *ciliata* and *T. sinensis* ; (c) $K_{a}/K_{s}$ of the orthologous genes between *T. ciliata* var. *ciliata* and *T. sinensis* , between *T. ciliata* var. *ciliata* and *A. thaliana* , and between *T. sinensis* and *A. thaliana* .

The distribution of synonymous substitution rate ($K_{s}$) between paralogous genes indicated that MADS‐box genes underwent three duplication events in both *T. ciliata* var. *ciliata* and *T. sinensis* (Figure 8a). Figure 8b indicated that most gene family members were under purifying selection during evolution ($K_{a}/K_{s}$ < 1). The $K_{a}/K_{s}$ ratio ranged from 0.0311 to 1.0425 in *T. ciliata* var. *ciliata*, with the mean of 0.2549 ± 0.1934 from estimates of 100 gene members, and from 0.0413 to 0.8141 in *T. sinensis* , with the mean of 0.3283 ± 0.1981 from estimates of 63 gene members. Kolmogorov–Smirnov (K‐S) test showed a significant difference between two species (statistics *D* = 0.2436, *p* value = 0.0163), indicating that *T. ciliata* var. *ciliata* was generally under stronger purifying selection than *T. sinensis* among the MADS‐box gene family members.

The $K_{a}/K_{s}$ ratio for orthologous MADS‐box genes ranged from 0.0042 to 0.3936 between *T. ciliata* var. *ciliata* and *A. thaliana* , with the mean of 0.1400 ± 0.0707 from estimates of 101 gene members, and from 0.0020 to 0.3859 between *T. sinensis* and *A. thaliana*, with the mean of 0.1526 ± 0.0762 from estimates of 72 gene members (Figure 8c). There were no significant differences between these two pairs of comparisons (K‐S test, *D* = 0.1225, *p* value = 0.5013). This indicated that strong purifying selection occurred for all MADS‐box mutant genes in *Toona* species compared with those in *A. thaliana* , and more than 85% (1‐$K_{a}/K_{s}$) of deleterious mutant genes were eliminated by natural selection. However, selection strength was generally weaker for all MADS‐box mutant genes in *T. ciliata* var. *ciliata* compared with *T. sinensis* , with $K_{a}/K_{s}$ of 0.4081 ± 0.2947 and the range from 0.0289 to 1.8828. There were eight orthologous genes between *T. ciliata* var. *ciliata* and *T. sinensis* showing positive selection, and these genes belonged to the AGL32, Mβ, MIKC*, AG/STK, and AP1/FUL subfamilies.

### 2.6 Expression Comparison Among Taxa in the MADS‐Box Genes

**FIGURE 9:**
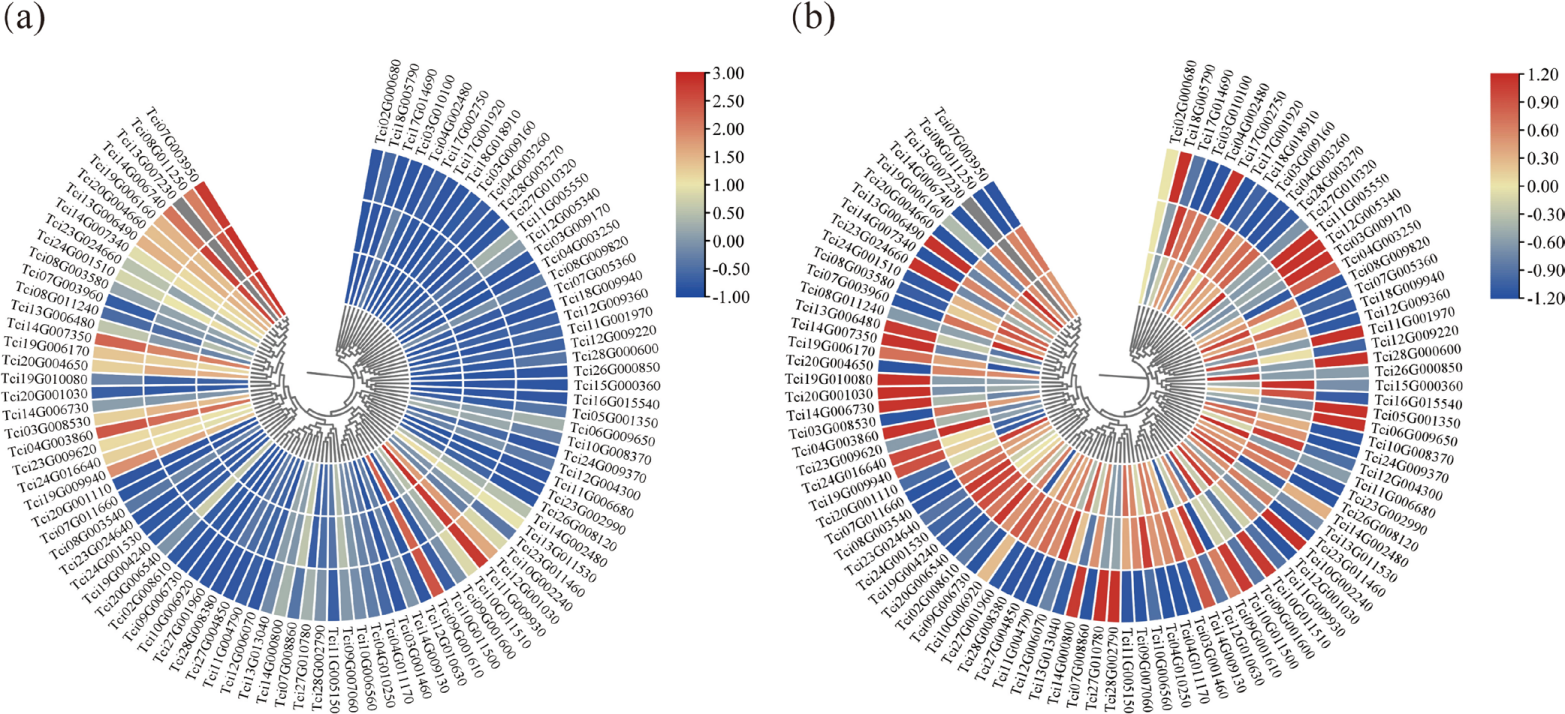
Heatmaps for expression profiles of the MADS‐box genes in flowers of 150 samples. (a) Comparison of expression levels among different genes for each taxon; (b) Comparison of expression levels among three taxa for each MADS‐box gene. In each figure, the outside circle represents the expression profile of *T. sinensis* , the middle circle for *T. ciliata* var. *pubescens*, and the innermost circle for *T. ciliata* var. *ciliata*. Levels of gene expression are indicated in different colors.

Based on the transcriptomic data from samples of mixed flowers of 150 individuals (Xiao 2025), we plotted a heatmap in terms of expression levels of the MADS‐box genes. Figure 9a shows the expression differences among 97 MADS‐box genes for each taxon. Figure 9b shows the expression differences among three taxa for each MADS‐box gene. The analysis revealed that the expression patterns of MADS‐box genes had certain commonalities among *T. ciliata* var. *ciliata*, *T. ciliata* var. *pubescens*, and *T. sinensis* , particularly within the SEP, AP1/FUL, AG/STK, PI, and AP3 subfamilies, where these genes exhibited relatively high expression levels. Notably, the expression patterns of these subfamily genes were highly conserved across species, indicating the conserved functions of the MADS‐box genes in floral development in *T. ciliata* var. *ciliata* and *T. sinensis* . Comparative expression analysis revealed that most MADS‐box genes exhibited similar patterns between *T. ciliata* var. *ciliata* and *T. ciliata* var. *pubescens*, likely reflecting their close phylogenetic relationship. However, the expression levels of many MADS‐box genes in *T. sinensis* varied, indicating the presence of different regulatory mechanisms in floral development. A reviewer pointed out the impact of growing niches on gene expression. Note that the large samples of flowers at the same developmental stage could mainly reflect genetic variation between species, although a complete removal of the influence of growing niches on interspecific variation in gene expression is challenging. The large sample sizes could provide robust inferences for TWAS (described in the next section) within each taxon.

**FIGURE 10:**
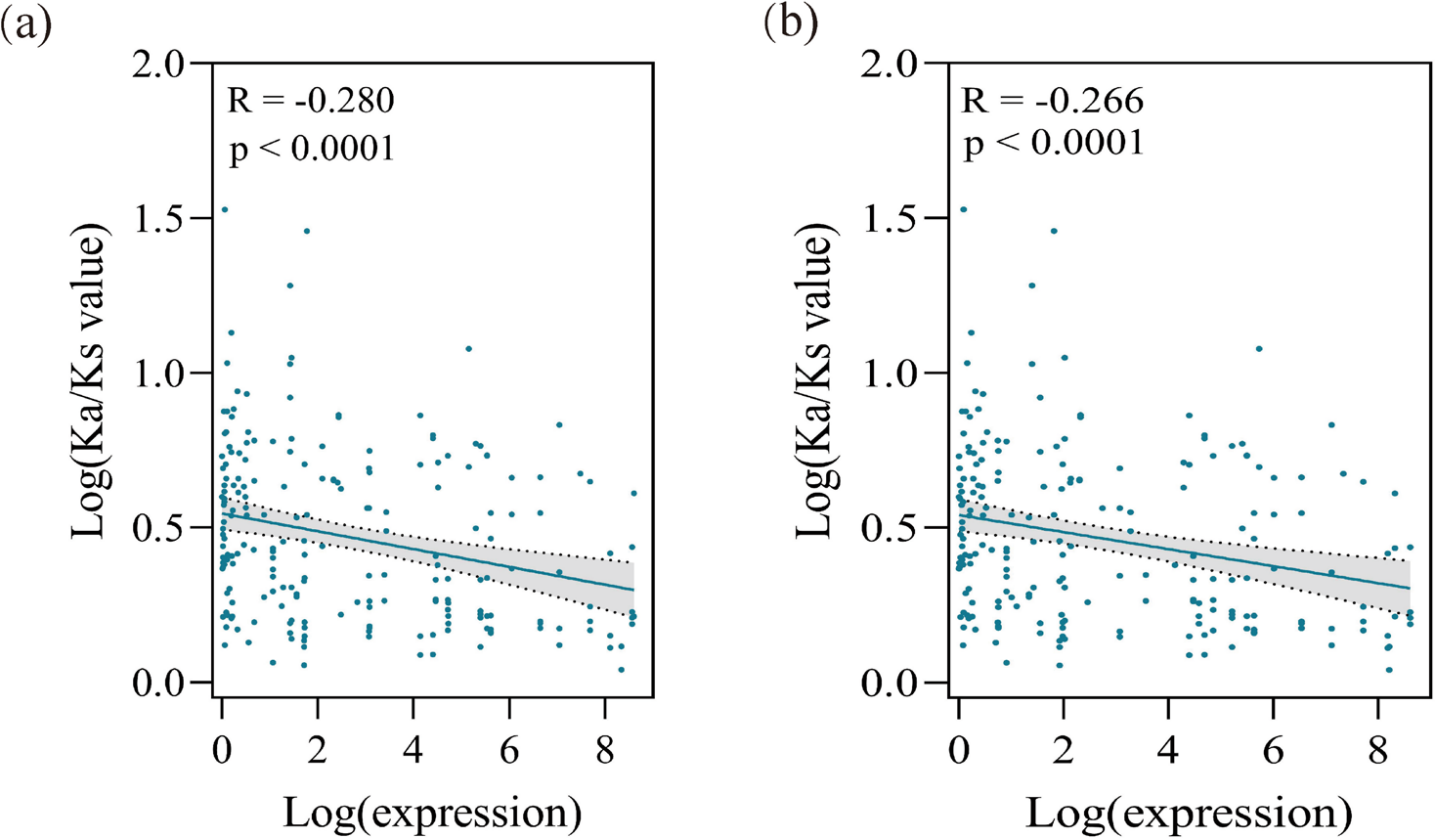
Correlation between the gene expression level and evolutionary rate ($K_{a}/K_{s}$). (a) *T. sinensis* — *T. ciliata* var. *ciliata*; (b) *T. sinensis* — *T. ciliata* var. *pubescens*. In both figures, $K_{a}/K_{s}$ values were calculated for orthologous gene pairs. Gene expression levels were obtained from transcriptome data of 100 flowers. A significantly negative correlation occurred between log‐transformed gene expression levels and log‐transformed $K_{a}/K_{s}$ values in both taxon pairs.

We further tested the relationship between the gene expression level and the evolutionary rate of MADS‐box genes. The MADS‐box genes from *A. thaliana* were employed as the referent genes to calculate the $K_{a}/K_{s}$ values for orthologous genes in *T. ciliata* var. *ciliata* (or *T. ciliata* var. *pubescens*) and *T. sinensis* . The expression levels of MADS‐box genes were derived from transcriptomes of flowers of 100 samples *T. sinensis* and *T. ciliata* var. *pubescens*. Figure 10 indicated the presence of significantly negative correlations between $K_{a}/K_{s}$ and gene expression levels. The deleterious mutant alleles underwent stronger purifying selection but had higher expression levels in flowers.

### 2.7 TWAS of the MADS‐Box Genes

**FIGURE 11:**
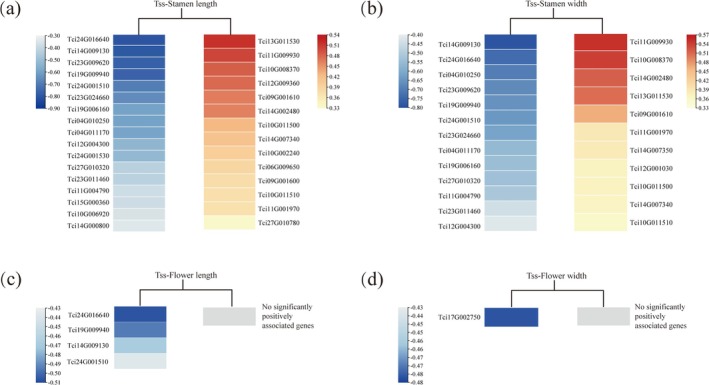
TWAS between gene expression and floral traits in *T. sinensis* . The MADS‐box genes significantly associated with four floral traits (FDR < 0.05) are presented: (a) stamen length, (b) stamen width, (c) flower length, and (d) flower width. In each panel, heatmaps display the correlation coefficients between the gene expression level and a floral trait value. Blue blocks stand for genes with negative correlations with the trait in different degrees, red blocks for genes with positive correlations in different degrees, and gray blocks for genes without positive correlations with the trait.

TWAS was separately conducted to identify MADS‐box genes associated with each floral trait in *T. ciliata* var. *ciliata*, *T. ciliata* var. *pubescens*, and *T. sinensis* . We identified 31, 24, 4, and 1 genes that were significantly associated with the stamen length, stamen width, flower length, and flower width (FDR < 0.05), respectively, in *T. sinensis* (Figure 11). Notably, the stamen length and width were both affected by the expression of multiple MADS‐box genes. Specifically, there were 17 MADS‐box genes that were negatively correlated with the stamen length and 14 genes that were positively correlated with the trait (Figure 11a). There were respectively 13 and 11 MADS‐box genes that were negatively and positively correlated with the stamen width (Figure 11b). Four MADS‐box genes were negatively correlated with the flower length but none were significantly positively associated with the trait (Figure 11c). Only one gene was negatively correlated with the flower width, and none was significantly positively associated with this trait (Figure 11d). Among the MADS‐box genes, four genes, encompassing Tci24G016640 and Tci19G009940 (AG/STK subfamily), Tci14G009130 (AGL32 subfamily), and Tci24G001510 (SEP/AGL6 subfamily), were simultaneously significantly associated with the stamen length, stamen width, and flower length in *T. sinensis* , highlighting their important roles in floral trait regulation.

Although the MADS‐box genes associated with floral traits were also detected in *T. ciliata* var. *ciliata* and *T. ciliata* var. *pubescens*, none passed the FDR threshold after multiple‐test corrections (Table S2).

## 3 Discussion

The MADS‐box genes encode an array of transcription factors that are widely involved in various stages of plant growth and development and particularly play key roles in inflorescence, flowering, and fruit development (Becker and Theißen 2003). Throughout plant evolution, numerous gene duplication and loss events occurred in history. The retention of duplicated genes varies greatly across species, leading to significant differences in the richness and abundance of the MADS‐box genes among species. For instance, there are 107 MADS‐box gene members in *A. thaliana* (Parenicova et al. 2003), 108 members in *chrysanthemum* (Won et al. 2020), and 79 members in *Prunus persica* (L.) Batsch (Wells et al. 2015). Here we identified that more MADS‐box genes occurred in *T. ciliata* var. *ciliata* than in *T. sinensis* (97 vs. 75), consistent with a general pattern of more gene family expansion in *T. ciliata* var. *ciliata* (Wang, Xiao, He, Li, Lv, et al. 2022; Wang, Xiao, He, Li, Song, et al. 2022). The MADS‐box genes in the two species included both Type I and Type II of MADS‐box genes, and Type I was further subdivided into three subfamilies and Type II into 13 subfamilies. In *T. ciliata* var. *ciliata*, the MADS‐box genes were distributed in all subfamilies, while in *T. sinensis* , they were distributed in all subfamilies except for the FLC subfamily.

Compared with *A. thaliana* , both *T. ciliata* var. *ciliata* and *T. sinensis* had fewer Type I genes but more Type II genes. This evolutionary divergence is likely related to natural adaptation where the *Toona* species have a more restrictive distribution than *A. thaliana* . Notably, *A. thaliana* contains six genes in the FLC subfamily, while *T. ciliata* var. *ciliata* and *T. sinensis* have only one and zero *FLC* genes, respectively. It is recognized that the *FLC* genes in *A. thaliana* are critical regulators of flowering time and play crucial roles in ecological responses to cold stress. The *FLC* genes act as key integrators of environmental and endogenous developmental signals (Becker and Theißen 2003; Sheldon et al. 2000). Therefore, the *FLC* gene expansion facilitates adaptation to diverse environmental conditions (e.g., light and temperature) in different habitats. For instance, compared with *A. thaliana* and *Oryza sativa* , the FLC subfamily is significantly expanded in *Triticum aestivum* , which enhances its adaptation to various habitats and expands its global distribution (Schilling et al. 2020). In contrast, plants (e.g., *O. sativa* and *Citrullus lanatus* ) do not require vernalization for flowering and lack FLC subfamily genes (Arora et al. 2007; Wang et al. 2019). Thus, it is speculated that *T. ciliata* and *T. sinensis* could not require vernalization for flowering.

Structural differences in subfamilies of the MADS‐box genes between *T. ciliata* var. *ciliata* and *T. sinensis* highlight the divergent evolutionary processes underlying them. For instance, *T. ciliata* var. *ciliata* had 11 subfamilies of Type II members, while *T. sinensis* had eight subfamilies. It remains to be explored whether this difference limits the natural distribution of *T. ciliata* or not in comparison with *T. sinensis* in China. However, both *T. ciliata* var. *ciliata* and *T. sinensis* generally exhibited a simpler structure with fewer introns in Type I MADS‐box genes, which could arise from intron loss during gene evolution. In contrast, Type II genes were more complex and had abundant introns and considerable variation in arrangement, a pattern similar to that observed in the MADS‐box genes of *O. sativa* (Arora et al. 2007). Such a difference in intron abundance and arrangement implies the significance of these genes in structural evolution. The conserved motifs between *T. ciliata* var. *ciliata* and *T. sinensis* imply their effects on maintaining the conserved function of MADS‐box genes.

Gene duplication and transposition events are important factors driving the expansion of gene families and increasing the complexity of eukaryotic genomes (Hughes 1994). The presence of numerous collinearity blocks suggests that the MADS‐box gene families potentially underwent multiple gene duplication events prior to the divergence between *T. ciliata* var. *ciliata* and *T. sinensis* (Wang, Xiao, He, Li, Lv, et al. 2022). The interspecific differences in abundance and MADS‐box structure were formed during the subsequent genomic evolution after speciation. The evolutionary processes for generating such divergences are unknown. These divergences could contribute to the functional diversification of MADS‐box genes in regulating interspecific variation in floral traits. Additionally, the functional regulation of MADS‐box genes is closely associated with cis‐regulatory elements in their promoters (Preston and Hileman 2013). The MADS‐box gene promoters in both *T. ciliata* var. *ciliata* and *T. sinensis* contain numerous cis‐regulatory elements related to light response and hormone signaling, implying that these genes could regulate growth, flowering, and adaptation to environmental changes.

Similar patterns of interspecific selection pressures ($K_{a}/K_{s}$) generally reflect the long‐distant genetic relationships between *T. ciliata* var. *ciliata* (or *T. ciliata* var. *pubescens*) and *A. thaliana* and between *T. sinensis* and *A. thaliana* in the MDAS‐box gene family. However, our results imply that *T. ciliata* var. *ciliata* and *T. sinensis* underwent different strengths of purifying selection against MADS‐box genes after speciation. The interspecific selection pressures between *T. ciliata* and *T. sinensis* (0.4081 ± 0.2947) were generally weaker than interfamily members within *T. ciliata* (0.2549 ± 0.1934) or *T. sinensis* (0.3283 ± 0.1981), implying more strict limitations on the number of MADS‐box genes. The presence of eight orthologous genes between *T. ciliata* and *T. sinensis* with positive selection ($K_{a}/K_{s}$ > 1) suggests potentially disruptive selection driving species divergence in the AGL32, Mβ, MIKC*, AG/STK, and AP1/FUL subfamilies.

The identified subfamilies with genes of positive selection are likely related to plant mating systems besides their effects on adaptation to environments. Previous studies showed that the AGL32 (Bsister) subfamily with genes of positive selection affected ovule and fruit development in plants (Yang et al. 2012; Yin and Xue 2012). The AGL32 subfamily gene *MADS‐55* affected self‐incompatibility in *T. kok‐saghyz* (self‐incompatibility) and *T. mongolicum* (apomixis) (Chen et al. 2023). The MIKC* subfamily genes affect pollen maturation in *Arabidopsis* and rice (Liu et al. 2013). Class C gene *AGAMOUS* (*AG*) affects stamen and carpel identity during early flower development and is closely associated with the formation of carpels, ovules, and fruit (Bowman 1989). The D‐class gene *SEEDSTICK* (*STK*) is involved in the development of ovules and seeds, and in promoting seed dispersal after fertilization, with strong temporal and spatial expression specificity (Dreni and Kater 2014; Mizzotti et al. 2012). In *Arabidopsis thaliana* , the AP1/FUL subfamily includes *APETALA*1 (*AP*1), which functions as a class A gene in sepal and petal identity, and *FRUITFULL* (*FUL*), which is involved in the development of cauline leaves and siliques (Gu et al. 1998; Litt and Irish 2003). Therefore, it is naturally hypothesized that these subfamilies' genes are likely associated with divergent mating systems between *T. ciliata* and *T. sinensis* (Zhou et al. 2020). Note that *T. ciliata* and *T. sinensis* are not an ideal model system for testing this hypothesis since their mating systems are not completely contrasted (outcrossing for *T. sinensis* vs. predominantly outcrossing with partial selfing and inbreeding for *T. ciliata* ). Future study using alternative plant species with contrast mating systems (e.g., selfing vs. outcrossing) could be effective to verify whether these MADS‐box genes evolve plant mating systems.

Previous research indicated a significantly negative correlation between the $K_{a}/K_{s}$ ratio and gene expression level (Li, Li, et al. 2023). This was the same case for most MADS‐box genes in *T. ciliata* and *T. sinensis* where a significant negative correlation between $K_{a}/K_{s}$ values and gene expression levels occurred. The pattern is consistent with theoretical prediction where, given a deleterious mutant allele with section coefficient *s*, the $K_{a}/K_{s}$ ratio decreases as the purifying selection strength ($4N_{e}s$ where *N*_*e*_ is the effective population size) increases (Kimura 1962; Li, Xiao, et al. 2023; Xiao et al. 2024). This result further suggests a complex interplay among selection pressure, evolutionary rates, and gene expression in MADS‐box genes, with these factors jointly shaping floral development of *T. ciliata* and *T. sinensis* .

Patterns of expression profiles of MADS‐box family genes revealed significant differences in flowers between *T. ciliata* and *T. sinensis* . Notably, genes from SEP, AP1/FUL, AG/STK, PI, and AP3 subfamilies exhibited unregulated expression, suggesting that these genes affected flowering and floral organ morphogenesis in both species. The differential expression of these genes was associated with interspecific variations in floral traits and likely related to divergent mating systems between *T. ciliata* and *T. sinensis* . Furthermore, MADS‐box family genes are key regulators during floral development in *T. ciliata* and *T. sinensis* . TWAS also evidenced the correlations between MADS‐box genes and floral trait variations. These findings provide new insights into future studies on the functional roles of MADS‐box genes in driving interspecific variation in floral traits.

TWAS in *T. sinensis* revealed significant associations between the expression of some MADS‐box genes and floral traits, particularly the stamen length and width, indicating that these genes could involve floral development. Four genes (Tci24G016640, Tci19G009940, Tci14G009130, and Tci24G001510) were significantly associated with multiple traits, suggesting their core regulatory functions. Given that these floral traits are related to the plant mating system (Goodwillie et al. 2006; Lin and Ritland 1997), these findings imply that MADS‐box genes could participate in the evolution or maintenance of reproductive strategies. Reasons for no significant associations detected in *T. ciliata* var. *ciliata* or *T. ciliata* var. *pubescens* were likely due to the limited sample size and low phenotypic variation. Overall, the strong association between MADS‐box gene expression and floral traits in *T. sinensis* highlights the potential role of this gene family not only in floral development but also in shaping the mating system in *Toona* species.

One attention in the preceding discussions is concerned with the functions of the identified MADS‐box genes associated with floral traits. Although Type I errors were strictly controlled (FDR < 5%) in TWAS, experimental verification of these gene functions is important in future studies. Both reviewers suggested future experiments to validate these findings, such as gene knockout and overexpression tests of a few identified MADS‐box genes by qPCR. Thus, separate experimental studies are needed in future studies to test gene functions with *T. ciliata* and *T. sinensis* , although some identified genes were tested in other plant species discussed above.

## 4 Materials and Methods

### 4.1 Data Acquisition

We collected the genomic data of *T. ciliata* var. *ciliata* from our previous study, which is accessible in the CNGB Nucleotide Sequence Archive (Accession number: CNP0001985) (Wang, Xiao, He, Li, Song, et al. 2022). The genomic data of *T. sinensis* were also retrieved from the CNGB Nucleotide Sequence Archive (Accession number: CNP0000958) (Ji et al. 2021). One reviewer suggested inclusion of closely related species for analysis. Although genome sequences of several species in the Meliaceae family are available, including *Azadirachta indica* [NCBI SRA 053330] (Krishnan et al. 2012), *Xylocarpus granatum* (GenBank accession: GCA_019650275.1) and *Toona fargesii* A. Chev. (Ma et al. 2025), gene annotations (gff files) were not publicly available. These species were not included for MADS‐box gene family analysis. The amino acid sequences of MADS‐box gene family members from *A. thaliana* (a model plant) were downloaded from the TAIR website (https://www.arabidopsis.org/).

Transcription data (RNA‐seq) were also based on our recent study (Xiao 2025), including 150 samples of mixed flowers that were at the same developmental stages (mainly mature flowers). We collected flower samples from 50 trees of *T. ciliata* var. *ciliata* and 50 trees of *T. ciliata* var. *pubescens* in Lijiang and Dali cities in Yunnan Province, respectively. One reviewer concerned the fluctuation of gene expression with time. We acknowledged this variation in transcriptomic analysis and collected all flower samples at the same developmental stages within 2 days at each location. Note that the field sampling was challenging since the trees were tall trees (> 20 m) at the reproductive period (more than 10 years old). The grow habitats in the two locations in Yunnan Province are similar, with the annual average temperature of approximately 16°C–19°C, the total annual rainfall of about 800–1100 mm, and the dominant red soil (Cai 2016; Du et al. 2016; Li 2021). Samples of mixed flowers from 50 trees of *T. sinensis* were collected in the same way in Ziyang and Suining cities, Sichuan Province. The two grow habitats are also similar, with the annual temperature of about 17.5°C and the total annual rainfall of about 900–1100 mm, but diverse soils (dominant tide sand soil in Zijiang and dominant purple soil in Suining) (Huang 2011; Meng 2013). The four localities generally had comparable habitats. The sampled trees were located more than 100 m away from each other to reduce potentially genetic relatedness in natural forests. We identified two varieties of *T. ciliata* based on leaf morphological characteristics according to previous studies (Chen et al. 1997; Zhang 2018). A sample of mixed mature flowers from each tree was immediately stored at −80°C in a cryogenic refrigerator for RNA extraction.

For each sampled tree, we measured seven floral traits of mature flowers, including the flower opening width (OW), flower length (FL), flower width (FW), stamen length (or filament length, SL), pistil length (or style length, PL), stamen width (or anther length, SW), and pistil width (or stigma width, PW). Thirty mature flowers were randomly collected from each sampled tree to measure floral traits. One hundred and fifty individuals were measured using a digital caliper, with 4500 flowers phenotyped in total. Floral traits from 1500 mature flowers were measured within 3 days in each location. Means of floral traits from 30 flowers of each tree were calculated to represent the tree flower characters (50 individual samples for each taxon) for TWAS.

One hundred and fifty samples were entrusted to Beijing Biomarker Technology Co. Ltd. for RNA‐seq analysis. The technique details for RNA‐seq were provided in (Xiao 2025). Gene expression was measured in terms of FPKM (fragments per kilobase of transcript per million fragments mapped).

### 4.2 Identification and Structure Analysis of the MADS‐Box Genes

Hidden Markov Model (HMM) files corresponding to two protein domains, SRF‐TF (PF00319) and K‐box (PF01486), of the MADS‐box gene family were downloaded from the PFAM database (http://pfam‐legacy.xfam.org/) (Mistry et al. 2021). Using the HMMER 3.0 software (Potter et al. 2018), we searched for the protein sequences of *T. ciliata* var. *ciliata* and *T. sinensis* with default parameters. The MADS‐box protein sequences from *A. thaliana* were downloaded from the TAIR website (https://www.arabidopsis.org/), and the obtained sequences were filtered and validated using Blast 2.16.0 (McGinnis and Madden 2004) in combination with the NCBI CDD (Lu et al. 2020). The physicochemical properties of the MADS‐box proteins from *T. ciliata* var. *ciliata* and *T. sinensis* were analyzed using the ExPASy online tool (https://web.expasy.org/protparam/) (Duvaud et al. 2021). Additionally, the subcellular localization of these proteins was predicted using the Cell‐PLoc 2.0 website (http://www.csbio.sjtu.edu.cn/bioinf/Cell‐PLoc/).

The conserved domains of MADS‐box proteins from *T. ciliata* var. *ciliata* and *T. sinensis* were analyzed using the MEME 5.5.7 online tool (http://meme‐suite.org/), setting a limit of 10 conserved motifs (Bailey et al. 2009). Exon and intron information for the MADS‐box genes in *T. ciliata* var. *ciliata* and *T. sinensis* was extracted from the respective genomic GFF files. Conserved protein domains of the MADS‐box gene family members in *T. ciliata* var. *ciliata* and *T. sinensis* were analyzed using the Batch CD Search function on the NCBI website (https://www.ncbi.nlm.nih.gov/Structure/bwrpsb/bwrpsb.cgi). The conserved protein domains and gene structures of the MADS‐box gene family were then visualized using TBtools 2.210 (Chen et al. 2020).

The chromosomal distribution of MADS‐box genes in *T. ciliata* var. *ciliata* and *T. sinensis* was visualized using TBtools 2.210 (Chen et al. 2020), based on the gene location information from the GFF annotation files. Intra‐ and interspecies collinearity analyses of the MADS‐box gene family members from *T. ciliata* var. *ciliata*, *T. sinensis* , and *A. thaliana* were conducted using the One Step MCScanX–Super Fast tool in the TBtools package.

The upstream sequences of 2000 bp from the transcriptional start site (TSS) of MADS‐box genes in *T. ciliata* var. *ciliata* and *T. sinensis* were extracted using TBtools 2.210 as the promoter regions. Cis‐regulatory elements within these promoter sequences were predicted using the PlantCARE online tool (http://bioinformatics.psb.ugent.be/webtools/plantcare/html/) (Lescot et al. 2002). The results were statistically analyzed, and heatmaps and stacked bar charts were generated to visualize the distribution of these elements.

Using the protein sequences of the MADS‐box gene family in *A. thaliana* as a reference, protein–protein interaction (PPI) networks among the MADS‐box family members in *T. ciliata* var. *ciliata* and *T. sinensis* were predicted using the STRING 12.0 online database (https://cn.string‐db.org/).

### 4.3 Assessment of the Evolution of the MADS‐Box Gene Family

Protein sequences of *T. ciliata* var. *ciliata* and *T. sinensis* were aligned with those of *A. thaliana* using MAFFT 7.490 (Katoh and Standley 2013). The aligned sequences were then trimmed using TrimAl 1.5 with default parameters (Capella‐Gutiérrez et al. 2009). A phylogenetic tree was constructed using the IQ‐TREE 2.0.7 software (https://iqtree.github.io/), with 1000 bootstrap repetitions and automatic thread settings, while other parameters were set as defaults (Bui Quang et al. 2020). The resulting phylogenetic tree file was visualized using the iTOL V4 online tool (https://itol.embl.de/itol.cgi) (Letunic and Bork 2019). Based on the phylogenetic relationships of the MADS‐box proteins, the MADS‐box family members were classified.

The CDS sequences of MADS‐box gene pairs among *T. ciliata* var. *ciliata*, *T. sinensis* , and *A. thaliana* were prepared and aligned, then converted into axt format. The synonymous substitution rate ($K_{s}$) and nonsynonymous substitution rate ($K_{a}$) and their ratio ($K_{a}/K_{s}$) were calculated using the YN (Yang‐Nielsen) model with KaKs_calculator 3.0 (Zhang 2022). Density plots were generated using the ggplot2 package in R (Villanueva and Chen 2019), and violin plots were created using GraphPad Prism 8.

Spearman correlation was analyzed using SPSS 22.0 to test the relationship between the evolutionary rate ($K_{a}/K_{s}$) and expression levels of the MADS‐box genes in *T. ciliata* var. *ciliata* and *T. sinensis* . Scatter plots were generated using GraphPad Prism 8 to depict this relationship.

### 4.4 TWAS of the MADS‐Box Genes

FPKM values for MADS‐box genes were obtained from the RNA‐seq data of 150 samples of mixed flowers of *T. ciliata* var. *ciliata*, *T. ciliata* var. *pubescens*, and *T. sinensis* . Heatmaps for the expression of the MADS‐box genes in *T. ciliata* var. *ciliata* and *T. sinensis* were plotted using TBtools 2.210. Pearson's correlation was tested for each floral trait (inner flower width, flower width, flower length, stamen length, stamen width, pistil length, and pistil width) with the expression levels of MADS‐box genes in *T. ciliata* var. *ciliata*, *T. ciliata* var. *pubescens*, and *T. sinensis* . The FDR (false discovery rate) of less than 0.05 was set as a significant level.

## Author Contributions

**Xiao‐Han Liu:** conceptualization (equal), methodology (equal), resources (equal), software (lead), visualization (lead), writing – original draft (lead). **Yu Xiao:** data curation (equal), resources (equal). **Yan‐Wen Lv:** resources (equal). **Zi‐Yun Wang:** resources (equal). **Chao Wu:** resources (equal). **Hui Xie:** resources (equal). **Xin‐Sheng Hu:** conceptualization (equal), funding acquisition (lead), writing – review and editing (lead).

## Conflicts of Interest

The authors declare no conflicts of interest.

## Supporting information


**Table S1:** Physicochemical properties of MADS‐box proteins in *Toona ciliata* and *Toona sinensis*.

**Table S2:** Pearson's correlations and tests between the expression levels of MADS‐box genes and measurements of seven floral traits: (A) *T. ciliata* var. ciliata; (B) *T. ciliata* var. pubescens; (C) *T. sinensis* .

## Data Availability

The genome sequence data were downloaded from https://db.cngb.org/search/project/CNP0001985/ for *Toona ciliata* and https://db.cngb.org/search/project/CNP0000958/ for *Toona sinensis*. Phenotypic (floral traits) and transcriptomic data of 150 individuals were available on request from XSH.
