# Supplementary material for: Assessing Evolutionary Divergence in Genome‐Wide MADS‐Box Genes and Expression Profiles Between Toona ciliata and Toona sinensis
Source: Ecol Evol. 2025 Oct 13;15(10):e72328. doi: 10.1002/ece3.72328 (PMC12516090; doi:10.1002/ece3.72328)
Supplement: Supplementary file 1 — Table S1: Physicochemical properties of MADS‐box proteins in Toona ciliata and Toona sinensis. [file ECE3-15-e72328-s001.docx]

**Assessing evolutionary divergence in genome-wide MADS-box genes and expression profiles between *Toona ciliata* and *Toona sinensis***

Xiao-Han Liu^1,2^, Yu Xiao^1,2^, Yan-Wen Lv^1,2^, Zi-Yun Wang^1,2^, Chao Wu ^1,2^, Hui Xie^1,2^, Xin-Sheng Hu^1,2*^

1. College of Forestry and Landscape Architecture, South China Agricultural University, Guangzhou 510642, China

2. Guangdong Key Laboratory for Innovative Development and Utilization of Forest Plant Germplasm, Guangzhou 510642, China

^*^ Correspondence: xinsheng@scau.edu.cn

Table S1. The physicochemical properties of MADS-box proteins in *Toona ciliata* and *Toona sinensis*

| Sequence ID | Number of Amino Acid | Molecular Weight | pI | Instability Index | Aliphatic Index | Grand Average of Hydropathicity | Subcellular localization |
| --- | --- | --- | --- | --- | --- | --- | --- |
| Tci02G000680 | 201 | 23009.03 | 5.27 | 41.89 | 76.67 | -0.589 | Nucleus |
| Tci02G008610 | 121 | 13874.11 | 6.13 | 52.29 | 109.42 | -0.335 | Nucleus |
| Tci03G001460 | 237 | 27957.53 | 6.35 | 61.86 | 80.68 | -0.819 | Nucleus |
| Tci03G008530 | 241 | 27483.22 | 9.76 | 57.99 | 77.72 | -0.755 | Nucleus |
| Tci03G009160 | 202 | 22515.69 | 9.07 | 52.56 | 63.81 | -0.638 | Nucleus |
| Tci03G009170 | 179 | 20322.49 | 9.73 | 46.49 | 86.59 | -0.602 | Nucleus |
| Tci03G010100 | 233 | 27149.39 | 4.65 | 62.97 | 65.67 | -0.87 | Nucleus |
| Tci04G002480 | 176 | 20070.87 | 7.54 | 63.37 | 68.64 | -0.691 | Nucleus |
| Tci04G003250 | 179 | 20287.44 | 9.49 | 47.47 | 84.41 | -0.616 | Nucleus |
| Tci04G003260 | 202 | 22323.42 | 9.07 | 46.93 | 67.72 | -0.554 | Nucleus |
| Tci04G003860 | 241 | 27678.35 | 9.65 | 64.35 | 76.51 | -0.788 | Nucleus |
| Tci04G010250 | 266 | 31167.28 | 6.14 | 67.62 | 88.01 | -0.694 | Nucleus |
| Tci04G011170 | 254 | 29720.7 | 6.35 | 62.41 | 86.42 | -0.707 | Nucleus |
| Tci05G001350 | 349 | 38396.55 | 8.8 | 53.8 | 69.03 | -0.57 | Nucleus |
| Tci06G009650 | 355 | 39266.49 | 7.69 | 52.96 | 69.46 | -0.564 | Nucleus |
| Tci07G003950 | 243 | 27870.76 | 8.88 | 41.1 | 80.7 | -0.673 | Nucleus |
| Tci07G003960 | 250 | 28969.95 | 8.55 | 59.54 | 82.64 | -0.721 | Nucleus |
| Tci07G005360 | 212 | 24155.69 | 8.96 | 30.99 | 74.58 | -0.692 | Nucleus |
| Tci07G008860 | 229 | 26268.95 | 6.49 | 60.16 | 86.42 | -0.693 | Nucleus |
| Tci07G011660 | 210 | 24535.11 | 8.79 | 67.64 | 83.1 | -0.733 | Nucleus |
| Tci08G003540 | 203 | 23661.36 | 9.07 | 61.66 | 77.29 | -0.681 | Nucleus |
| Tci08G003580 | 246 | 27888.75 | 8.31 | 45.48 | 94.35 | -0.551 | Nucleus |
| Tci08G009820 | 268 | 30381.59 | 9.2 | 44.62 | 75.3 | -0.885 | Nucleus |
| Tci08G011240 | 250 | 29183.38 | 9.26 | 63.99 | 85.4 | -0.696 | Nucleus |
| Tci08G011250 | 243 | 28022.02 | 8.56 | 40.41 | 85.1 | -0.612 | Nucleus |
| Tci09G001600 | 241 | 28059.19 | 8.86 | 61.03 | 85.39 | -0.69 | Nucleus |
| Tci09G001610 | 212 | 24826.33 | 8.76 | 63.81 | 84.62 | -0.857 | Nucleus |
| Tci09G006730 | 226 | 25338.12 | 6.98 | 65.82 | 97.92 | -0.454 | Nucleus |
| Tci09G007060 | 237 | 27359.4 | 9.56 | 48.46 | 87.59 | -0.732 | Nucleus |
| Tci10G002240 | 209 | 24128.42 | 9.12 | 52.29 | 82.06 | -0.782 | Nucleus |
| Tci10G006560 | 186 | 21636.98 | 9.59 | 43.94 | 89.57 | -0.667 | Nucleus |
| Tci10G006920 | 228 | 25343.1 | 6.78 | 56.03 | 91.49 | -0.42 | Nucleus |
| Tci10G008370 | 407 | 45914.83 | 5.06 | 54.69 | 79.56 | -0.502 | Nucleus |
| Tci10G011500 | 150 | 16949.74 | 9.51 | 38.71 | 102.07 | -0.254 | Nucleus |
| Tci10G011510 | 218 | 25694.45 | 9.3 | 64.05 | 87.71 | -0.844 | Nucleus |
| Tci11G001970 | 319 | 37360.01 | 8.75 | 48.99 | 63.61 | -0.632 | Nucleus |
| Tci11G004790 | 228 | 25532.79 | 6.47 | 51.39 | 86.01 | -0.653 | Nucleus |
| Tci11G005150 | 225 | 25975.47 | 7.78 | 41.31 | 88.76 | -0.744 | Nucleus |
| Tci11G005550 | 172 | 18730.74 | 9.03 | 43.82 | 93.08 | -0.299 | Nucleus |
| Tci11G006680 | 289 | 32772.37 | 8.85 | 55.22 | 86.4 | -0.496 | Nucleus |
| Tci11G009930 | 223 | 25908.48 | 9.42 | 46.4 | 70.81 | -0.796 | Nucleus |
| Tci12G001030 | 223 | 25782.11 | 9.31 | 41.38 | 67.8 | -0.867 | Nucleus |
| Tci12G004300 | 325 | 37064.89 | 6.33 | 56.82 | 81.29 | -0.488 | Nucleus |
| Tci12G005340 | 199 | 22032.13 | 6.6 | 42.97 | 77.54 | -0.552 | Nucleus |
| Tci12G006070 | 228 | 25899.55 | 6.94 | 65.49 | 85.96 | -0.601 | Nucleus |
| Tci12G009220 | 327 | 38303.14 | 8.92 | 46.28 | 66.24 | -0.613 | Nucleus |
| Tci12G009360 | 327 | 38286 | 8.75 | 48.12 | 65.66 | -0.668 | Nucleus |
| Tci12G010630 | 318 | 36380.77 | 6.12 | 48.86 | 76.38 | -0.804 | Nucleus |
| Tci13G006480 | 244 | 28475.73 | 9.65 | 61.07 | 82.75 | -0.801 | Nucleus |
| Tci13G006490 | 246 | 28079.77 | 7.71 | 48.35 | 82.03 | -0.658 | Nucleus |
| Tci13G007230 | 253 | 28807.88 | 8.14 | 45.33 | 85.57 | -0.558 | Nucleus |
| Tci13G011530 | 365 | 41079.31 | 6.61 | 54.74 | 77.45 | -0.6 | Nucleus |
| Tci13G013040 | 255 | 28978.97 | 5.83 | 56.68 | 89.92 | -0.584 | Nucleus |
| Tci14G000800 | 249 | 28199.27 | 5.92 | 55.66 | 92.45 | -0.456 | Nucleus |
| Tci14G002480 | 350 | 39359.51 | 7.14 | 50.02 | 79.37 | -0.581 | Nucleus |
| Tci14G006730 | 193 | 21906.1 | 6.14 | 54.23 | 98.55 | -0.407 | Nucleus |
| Tci14G006740 | 245 | 28029.04 | 8.19 | 47.25 | 86 | -0.6 | Nucleus |
| Tci14G007340 | 247 | 28200.95 | 9.33 | 42.14 | 86.48 | -0.65 | Nucleus |
| Tci14G007350 | 243 | 28298.4 | 9.03 | 57.51 | 81.89 | -0.777 | Nucleus |
| Tci14G009130 | 236 | 27823.83 | 7.73 | 68.34 | 89.66 | -0.742 | Nucleus |
| Tci15G000360 | 239 | 27085.05 | 9.36 | 61.69 | 63.26 | -0.707 | Nucleus |
| Tci16G015540 | 240 | 27161.33 | 9.41 | 58.09 | 68.67 | -0.585 | Nucleus |
| Tci17G001920 | 287 | 32126.74 | 5.93 | 44.28 | 70.07 | -0.636 | Nucleus |
| Tci17G002750 | 136 | 14997.26 | 9.3 | 56.03 | 78.82 | -0.495 | Nucleus |
| Tci17G014690 | 193 | 22143.91 | 6.01 | 44.05 | 65.65 | -0.908 | Nucleus |
| Tci18G005790 | 224 | 25816.11 | 5.13 | 38.34 | 75.31 | -0.673 | Nucleus |
| Tci18G009940 | 299 | 34393.17 | 9.23 | 44.78 | 67.53 | -0.583 | Nucleus |
| Tci18G018910 | 390 | 43165.46 | 4.78 | 39.59 | 70.9 | -0.618 | Nucleus |
| Tci19G004240 | 207 | 24312.19 | 9.33 | 63.17 | 85.7 | -0.738 | Nucleus |
| Tci19G006160 | 253 | 28874.86 | 9.23 | 53.56 | 82.41 | -0.609 | Nucleus |
| Tci19G006170 | 246 | 28520.35 | 9.05 | 58.21 | 79.72 | -0.85 | Nucleus |
| Tci19G009940 | 177 | 20857.92 | 9.72 | 56.81 | 86.5 | -0.812 | Nucleus |
| Tci19G010080 | 202 | 23060.87 | 7.73 | 50.72 | 85.99 | -0.309 | Nucleus |
| Tci20G001030 | 202 | 23195.92 | 7.7 | 51.43 | 88.86 | -0.351 | Nucleus |
| Tci20G001110 | 254 | 28975.4 | 9.09 | 55.05 | 87.09 | -0.346 | Nucleus |
| Tci20G004650 | 246 | 28440.04 | 7.77 | 53.28 | 84.07 | -0.846 | Nucleus |
| Tci20G004660 | 255 | 28766.74 | 9.05 | 49.31 | 83.33 | -0.556 | Nucleus |
| Tci20G006540 | 219 | 25407.38 | 9.36 | 57.23 | 81.42 | -0.677 | Nucleus |
| Tci23G002990 | 202 | 22817.77 | 8.87 | 57.1 | 77.77 | -0.822 | Nucleus |
| Tci23G009620 | 253 | 29092.86 | 9.15 | 55.6 | 80.24 | -0.763 | Nucleus |
| Tci23G011460 | 201 | 22976.82 | 10.28 | 54.9 | 93.18 | -0.595 | Nucleus |
| Tci23G024640 | 219 | 25331.01 | 9.37 | 67 | 71.23 | -0.904 | Nucleus |
| Tci23G024660 | 244 | 27908.92 | 9.07 | 42.46 | 83.89 | -0.599 | Nucleus |
| Tci24G001510 | 244 | 27985.86 | 9.08 | 42.88 | 81.52 | -0.722 | Nucleus |
| Tci24G001530 | 230 | 26554.44 | 9.23 | 65.28 | 78 | -0.775 | Nucleus |
| Tci24G009370 | 323 | 37061 | 7.1 | 49.17 | 80.28 | -0.669 | Nucleus |
| Tci24G016640 | 233 | 27155.62 | 8.9 | 59.3 | 79.57 | -0.839 | Nucleus |
| Tci26G000850 | 205 | 23638.75 | 8.83 | 45.63 | 53.32 | -0.76 | Nucleus |
| Tci26G008120 | 119 | 13764.29 | 9.68 | 42.25 | 99.83 | -0.257 | Nucleus |
| Tci27G001960 | 213 | 24468.78 | 8.42 | 60.03 | 77.42 | -0.744 | Nucleus |
| Tci27G004850 | 214 | 24527.78 | 8.95 | 51.87 | 73.83 | -0.776 | Nucleus |
| Tci27G010320 | 196 | 22082.29 | 9.3 | 65.94 | 66.12 | -0.637 | Nucleus |
| Tci27G010780 | 244 | 28124.21 | 9.3 | 47.38 | 91.93 | -0.667 | Nucleus |
| Tci28G000600 | 346 | 40114.74 | 6.85 | 46.07 | 60 | -0.835 | Nucleus |
| Tci28G002790 | 244 | 27865.84 | 9.36 | 50.41 | 87.13 | -0.737 | Nucleus |
| Tci28G003270 | 207 | 23243.75 | 9.19 | 45.64 | 85.31 | -0.444 | Nucleus |
| Tci28G008380 | 212 | 24291.62 | 6.62 | 53 | 87.41 | -0.573 | Nucleus |
| Maker00013609 | 225 | 25852.12 | 5.33 | 44.66 | 71.11 | -0.67 | Nucleus |
| Maker00008113 | 170 | 19957.57 | 5.37 | 59.09 | 80.88 | -0.689 | Nucleus |
| Maker00022347 | 184 | 21232.95 | 8.87 | 63.06 | 76.3 | -0.763 | Nucleus |
| Maker00022402 | 179 | 20313.48 | 9.58 | 47.03 | 86.59 | -0.568 | Nucleus |
| Maker00022418 | 217 | 24224.48 | 7.94 | 51.91 | 65.25 | -0.715 | Nucleus |
| Maker00022459 | 85 | 9612.23 | 9.6 | 61.59 | 81.53 | -0.168 | Nucleus |
| Maker00022461 | 180 | 21075.2 | 5.89 | 59.7 | 73.61 | -0.708 | Nucleus |
| Maker00020199 | 209 | 24772.92 | 5.51 | 71.06 | 85.41 | -0.825 | Nucleus |
| Maker00023519 | 179 | 20163.21 | 9.52 | 44.78 | 82.79 | -0.649 | Nucleus |
| Maker00023619 | 170 | 19537.95 | 9.13 | 67.57 | 76.94 | -0.866 | Nucleus |
| Maker00023630 | 267 | 30645.81 | 5.33 | 61.92 | 67.9 | -0.666 | Nucleus |
| Maker00023696 | 217 | 24102.32 | 8.73 | 45.67 | 67.1 | -0.647 | Nucleus |
| Maker00032697 | 209 | 24772.92 | 5.51 | 71.06 | 85.41 | -0.825 | Nucleus |
| Maker00021711 | 351 | 38560.62 | 8.6 | 48.25 | 63.08 | -0.642 | Nucleus |
| Maker00007689 | 356 | 39024.16 | 8.25 | 52.53 | 68.74 | -0.533 | Nucleus |
| Maker00034150 | 181 | 20715.41 | 5.68 | 46.71 | 80.33 | -0.739 | Nucleus |
| Maker00034322 | 275 | 31046.45 | 9.25 | 32.22 | 70.58 | -0.888 | Nucleus |
| Maker00034456 | 163 | 18964.5 | 7.9 | 48.13 | 81.9 | -0.76 | Nucleus |
| Maker00003325 | 268 | 30287.48 | 9.26 | 49.73 | 72.8 | -0.894 | Nucleus |
| Maker00008157 | 124 | 14731.84 | 7.75 | 58.2 | 80.16 | -0.811 | Nucleus |
| Maker00008182 | 181 | 20734.5 | 5.68 | 43.87 | 80.33 | -0.672 | Nucleus |
| Maker00029632 | 98 | 11471.58 | 9.96 | 53.85 | 91.43 | -0.254 | Nucleus |
| Maker00031427 | 195 | 22244.64 | 5.19 | 52.43 | 104 | -0.29 | Nucleus |
| Maker00000277 | 164 | 19546.74 | 8.83 | 55.24 | 92.8 | -0.371 | Nucleus |
| Maker00000322 | 169 | 19938.89 | 7.08 | 54.85 | 90.59 | -0.811 | Nucleus |
| Maker00005931 | 114 | 13155.01 | 5.5 | 53.81 | 93.16 | -0.656 | Nucleus |
| Maker00006150 | 324 | 37552.34 | 9.6 | 40.7 | 91.48 | -0.517 | Nucleus |
| Maker00005551 | 217 | 25472.14 | 9.45 | 42.35 | 81.71 | -0.826 | Nucleus |
| Maker00002466 | 115 | 13270.79 | 4.8 | 36.72 | 91.48 | -0.895 | Nucleus |
| Maker00006324 | 318 | 37234.91 | 9.04 | 47.63 | 65.94 | -0.624 | Nucleus |
| Maker00009653 | 164 | 19266.65 | 5.33 | 47.69 | 87.93 | -0.973 | Nucleus |
| Maker00009704 | 203 | 22282.6 | 6.84 | 49.8 | 86.11 | -0.47 | Nucleus |
| Maker00031910 | 157 | 18319.58 | 6.91 | 50.7 | 72.68 | -0.854 | Nucleus |
| Maker00032084 | 147 | 17464.23 | 9.58 | 37.63 | 66.87 | -0.827 | Nucleus |
| Maker00032683 | 136 | 15267.89 | 4.96 | 50.8 | 81.03 | -0.908 | Nucleus |
| Maker00001824 | 324 | 37838.61 | 8.58 | 43.5 | 69.54 | -0.58 | Nucleus |
| Maker00026459 | 384 | 44379.42 | 5.99 | 47.35 | 59.64 | -0.815 | Nucleus |
| Maker00026531 | 138 | 15767.62 | 5.9 | 40.04 | 66.52 | -0.854 | Nucleus |
| Maker00026745 | 203 | 22205.22 | 6.23 | 39.99 | 73.6 | -0.58 | Nucleus |
| Maker00028620 | 324 | 37860.64 | 8.85 | 43.5 | 69.54 | -0.595 | Nucleus |
| Maker00013535 | 173 | 20510.1 | 5.73 | 68.39 | 83.41 | -0.966 | Nucleus |
| Maker00016673 | 191 | 21754.64 | 5.56 | 45.19 | 83.72 | -0.625 | Nucleus |
| Maker00016737 | 219 | 25645.11 | 6.02 | 64.52 | 74.43 | -0.965 | Nucleus |
| Maker00010752 | 190 | 21258.27 | 5.85 | 43.35 | 88.26 | -0.454 | Nucleus |
| Maker00031568 | 162 | 18567.05 | 5.96 | 47.31 | 83.09 | -0.775 | Nucleus |
| Maker00031621 | 133 | 15870.01 | 5.82 | 48.1 | 88.8 | -0.77 | Nucleus |
| Maker00029684 | 239 | 27148.18 | 9.42 | 59.95 | 64.48 | -0.683 | Nucleus |
| Maker00015777 | 240 | 27109.21 | 9.32 | 57.55 | 67.46 | -0.594 | Nucleus |
| Maker00000777 | 734 | 83918.82 | 5.32 | 42.37 | 93.3 | -0.151 | Nucleus |
| Maker00001842 | 439 | 50578.42 | 6.06 | 49.4 | 77.52 | -0.569 | Nucleus |
| Maker00001850 | 254 | 28134.37 | 8.59 | 43.51 | 70.71 | -0.523 | Nucleus |
| Maker00013783 | 128 | 13926.92 | 8.53 | 59.39 | 68.44 | -0.488 | Nucleus |
| Maker00013822 | 392 | 43424.71 | 4.64 | 42.46 | 71.02 | -0.652 | Nucleus |
| Maker00030721 | 238 | 27003.2 | 8.71 | 41.51 | 74.2 | -0.478 | Nucleus |
| Maker00000135 | 144 | 16550.61 | 5.57 | 52.66 | 85.42 | -0.876 | Nucleus |
| Maker00015002 | 140 | 16159.6 | 4.98 | 58.9 | 81.57 | -0.461 | Nucleus |
| Maker00015063 | 151 | 17656.3 | 8.69 | 60.7 | 94.97 | -0.451 | Nucleus |
| Maker00015386 | 127 | 14360.31 | 9.12 | 62.68 | 82.91 | -0.645 | Nucleus |
| Maker00015456 | 106 | 12381.56 | 5.17 | 73.81 | 98.4 | -0.122 | Nucleus |
| Maker00031027 | 191 | 21160.25 | 7.73 | 53.44 | 86.18 | -0.39 | Nucleus |
| Maker00031032 | 200 | 23148.1 | 5.62 | 45.99 | 90.3 | -0.782 | Nucleus |
| Maker00020888 | 291 | 33073.79 | 7.83 | 54.36 | 87.84 | -0.358 | Nucleus |
| Maker00021179 | 170 | 19497.96 | 6.32 | 46.85 | 90.12 | -0.708 | Nucleus |
| Maker00029113 | 195 | 22489.66 | 8.8 | 57.84 | 69.49 | -0.974 | Nucleus |
| Maker00029117 | 160 | 18280.8 | 6.92 | 43.07 | 84.06 | -0.654 | Nucleus |
| Maker00023809 | 185 | 20927.57 | 6.93 | 65.17 | 82.81 | -0.841 | Nucleus |
| Maker00023964 | 160 | 18271.69 | 6.43 | 43.64 | 80.44 | -0.738 | Nucleus |
| Maker00033560 | 211 | 24612.61 | 8.75 | 61.2 | 74.93 | -0.909 | Nucleus |
| Maker00019670 | 249 | 28521.06 | 9.64 | 36.95 | 62.65 | -0.717 | Nucleus |
| Maker00028473 | 152 | 17237.18 | 10.18 | 56.92 | 85.33 | -0.48 | Nucleus |
| Maker00003584 | 124 | 14103.08 | 6.74 | 38.6 | 88.79 | -0.872 | Nucleus |
| Maker00003745 | 221 | 24853.43 | 9.41 | 48.3 | 71.45 | -0.598 | Nucleus |
| Maker00033185 | 186 | 21710.7 | 8.77 | 50.9 | 77.53 | -0.931 | Nucleus |
| Maker00010054 | 221 | 24946.65 | 8.94 | 44.01 | 87.38 | -0.46 | Nucleus |
| Maker00006466 | 173 | 20619.24 | 5.33 | 71.17 | 87.92 | -0.953 | Nucleus |
